# Carcinoid heart disease: a guide for screening and timing of surgical intervention

**DOI:** 10.1007/s12471-017-1011-2

**Published:** 2017-06-19

**Authors:** E. A. Hart, T. A. Meijs, R. C. A. Meijer, K. M. Dreijerink, M. E. Tesselaar, C. A. de Groot, G. D. Valk, S. A. J. Chamuleau

**Affiliations:** 1 Department of Cardiology, University Medical Centre Utrecht, Utrecht, The Netherlands; 2 Department of Cardiothoracic Surgery, University Medical Centre Utrecht, Utrecht, The Netherlands; 3 Department of Endocrine Oncology, University Medical Centre Utrecht, Utrecht, The Netherlands; 4 Antoni van Leeuwenhoek Hospital, Amsterdam, The Netherlands; 5 Department of Cardiology, Slotervaart Medical Centre, Amsterdam, The Netherlands

**Keywords:** Carcinoid heart disease, Hedinger syndrome, Valvular disease, Screening, Surgery

## Abstract

The cardiac manifestations of a neuroendocrine tumour are referred to as carcinoid heart disease (CaHD) and are associated with a poor prognosis. Surgical intervention is the only proven therapeutic option and may prolong survival and quality of life. No consensus has been reached internationally with regard to screening for CaHD and the optimal timing for surgery. Although limited evidence is available on this matter, a trend towards early surgery and subsequent reduced mortality has been observed. In this review we provide an overview of the current understanding and propose a protocol to guide cardiologists in the screening for CaHD and the timing of referral to a specialised surgical centre.

## Introduction

### Neuroendocrine tumours

Well-differentiated neuroendocrine tumours (NETs) are rare malignancies, occurring in 5.25 per 100,000 people per year [1]. The majority of NETs develop in the small intestine, particularly in the ileum, and in the bronchopulmonary system. Less frequently NETs arise from other sites within the gastrointestinal tract, including colon, rectum and stomach [2]. Some of these tumours, in particular small intestinal NETs, secrete various vasoactive substances, including serotonin (5-hydroxytryptamine; 5‑HT), tachykinins, prostaglandins, histamine, and kallikrein. Typically, the liver inactivates these substances when released into the portal circulation. However, when a serotonin-producing NET metastasises to the liver, direct access to the systemic circulation may result in carcinoid syndrome. Carcinoid syndrome is characterised by episodic cutaneous flushing, hypotension, gut hypermotility with diarrhoea, and bronchospasms [3–5].

### Carcinoid heart disease

Patients with carcinoid syndrome are at risk to develop carcinoid heart disease (CaHD), also known as Hedinger syndrome. Since the introduction of somatostatin analogues, the incidence of CaHD has dropped from over 50% [6] to approximately 20% in patients with carcinoid syndrome [7]. CaHD is most likely caused by the paraneoplastic effects of vasoactive substances excreted by the tumour, particularly serotonin [5, 7, 8]. Although patients with CaHD are often asymptomatic in the early stages of the disease [9], signs of right heart failure are associated with disease progression.

The disease is characterised by plaque-like deposits of fibrous tissue involving the endocardium of the valve leaflets, cardiac chambers, and less frequently the intima of the pulmonary arteries and aorta [10]. Primarily the right side of the heart is affected, due to thickening and retraction of the tricuspid and pulmonary valve leaflets, with subsequent regurgitation and/or stenosis. Left-sided valve involvement occurs in less than 10% of patients with CaHD and is commonly observed in patients with a right-to-left shunt (e. g. patent foramen ovale) and elevated right heart-sided pressures, bronchial NETs or severe carcinoid syndrome with high amounts of vasoactive substances [6]. Sporadically, left-sided valvular disease is present in the absence of right-sided valve involvement [11, 12]. There is no clear explanation for the predominant right-sided valve involvement. Vasoactive substances excreted by the tumour are thought to be largely inactivated within the pulmonary circulation [13].

The presence of CaHD has a detrimental effect on the prognosis of NET patients and therefore early diagnosis and treatment, if possible, are of major importance [6, 14]. More specifically, the cause of death in CaHD patients is attributable to cardiac involvement in almost half of the cases [15].

In this review we present two case studies illustrating typical CaHD presentations. Next, we provide an overview of the current understandings and guidelines regarding CaHD. Additionally, a step-by-step approach is provided with regards to the screening, diagnosis, and surgical management.

## Diagnosis

### Biomarkers

Several biochemical markers are useful in the diagnosis of CaHD and are related to disease progression and prognosis. N‑terminal pro-brain natriuretic peptide (NT-proBNP) levels are significantly elevated in patients with CaHD compared with those without [16]. Due to its high sensitivity and specificity for the detection of CaHD in NET patients (92% and 91%, respectively), NT-proBNP may be useful as a screening test [16, 17]. Moreover, NT-proBNP levels are correlated with disease progression and survival [18].

High levels of chromogranin-A, a neuroendocrine secretory protein, are associated with the development of CaHD in NET patients [19] and with worse survival, especially when NT-proBNP levels are elevated.

5-Hydroxyindoleacetic acid (5-HIAA) is a metabolite of serotonin and its urinary excretion directly correlates with serotonin production. Urinary 5‑HIAA levels are significantly higher in NET patients with CaHD than in those without, and higher levels are associated with progression of cardiac involvement [9, 13]. Although specificity is low, suggesting the development and progression of CaHD is co-dependent on other factors [20], aggressive treatment to decrease 5‑HIAA levels is advisable.

### Imaging

Echocardiographic assessment is the gold standard for the detection of CaHD [21]. Two-dimensional and three-dimensional visualisation of fibrous plaques of the endocardium should be performed, as well as evaluation of wall thickness, wall motion abnormalities and right and left ventricular dimensions and function [21, 22]. More recently, the use of strain imaging (i. e. tissue Doppler imaging) has emerged as a helpful tool in the detection of early right ventricular (RV) dysfunction [23] and identification of high-risk patients [24].

Analysis of the pulmonary and tricuspid valve may reveal leaflet thickening with retraction and reduced mobility resulting in severe regurgitation, stenosis, or both [21, 22].

The presence of a patent foramen ovale may be detected through bubble or saline-contrast echocardiography. Myocardial carcinoid metastases are rare (4%) [6], primarily intramyocardial, and may be the only manifestation of CaHD [10]. On echocardiography these tumours can be identified by their homogeneous aspect and clearly defined contours.

RV size, function (ejection fraction) and regurgitant volumes are more accurately assessed using cardiac magnetic resonance (CMR) imaging. CMR imaging may therefore be a helpful tool when transthoracic echocardiography (TTE) is insufficient. Additionally, CMR imaging allows for careful assessment of myocardial tissue and may therefore aid in the detection of fibrous plaques and myocardial metastases [22, 25]. The typical features of CaHD are summarised in Table 1.

**Table 1:** Typical characteristics of carcinoid heart disease

Significant tricuspid regurgitation
Mixed pulmonary regurgitation and stenosis
Concomitant left-sided valve involvement (<10%), primarily in patients with persistent foramen ovale, bronchial carcinoid or severe carcinoid syndrome
Pathognomonic fibrous plaques on echocardiography involving the endocardium of valve leaflets and cardiac chambers
Intramyocardial metastases

Below, two typical CaHD cases are described with significant tricuspid regurgitation and intramyocardial metastases, respectively.

## Case report 1

**Fig. 1:**
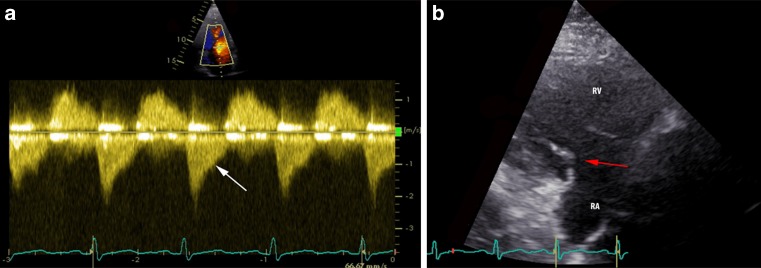
**a** Characteristic ‘dagger’ shaped jet on continuous wave Doppler **b** Parasternal view of RV inflow tract showing thickening and retraction of tricuspid leaflets (*arrow*) during systole. *RV* right ventricle, *RA* right atrium

A 47-year-old male with hepatic metastases of a NET of the ileum presented with progressive complaints of dyspnoea and hepatomegaly. TTE revealed severe tricuspid regurgitation with marked leaflet thickening, restriction of all three leaflets (Fig. 1), and a characteristic dagger-shaped jet on continuous wave Doppler (Fig. 1). Further assessment showed moderate RV dilation with mild RV dysfunction, mild pulmonary regurgitation, and mild mitral regurgitation based on leaflet thickening and retraction of the posterior leaflet. A patent foramen ovale was detected. Left ventricular (LV) function was normal. In anticipation of carcinoid progression of valve dysfunction the patient underwent successful tricuspid, mitral, and pulmonary valve replacement with bioprosthetic valves. The foramen ovale was closed. Postoperatively the RV function normalised and the tricuspid regurgitation was categorised as mild. Shortly thereafter the patient underwent successful resection of the primary tumour. Four months later the patient is in relatively good condition without signs of right heart decompensation and will be seen in the outpatient clinic for TTE in 3 months.

## Case report 2

**Fig. 2:**
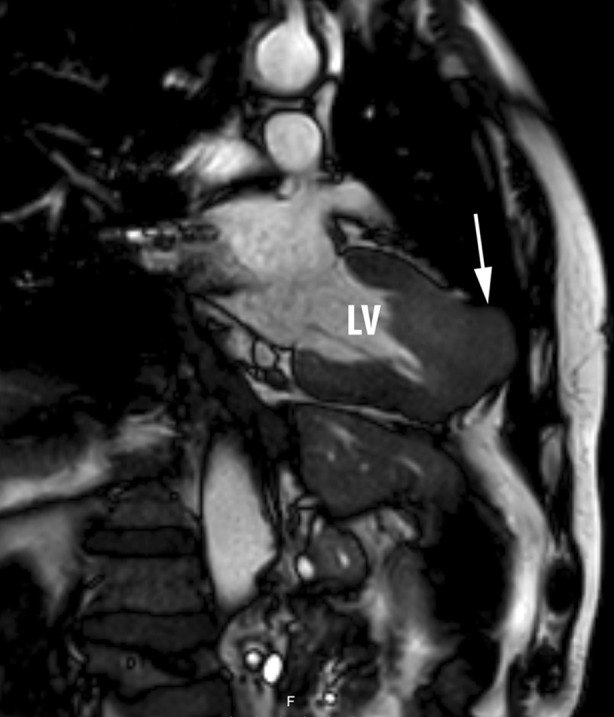
CMR image of one of the two apical intramyocardial lesions (*arrow*). *LV* left ventricle

A 74-year-old male with hepatic metastases of a NET of unknown origin was admitted to hospital with thoracic pain, suspicious for acute coronary syndrome, and fever. Electrocardiography revealed marked ST elevations in leads II, III, AVF, V2–V6, and negative T waves in the precordial leads. Further diagnosis ruled out myocardial ischaemia. No clinical signs of heart failure were present and TTE showed no valvular abnormalities. CMR imaging revealed two apical intramyocardial lesions (Fig. 2). The patient was diagnosed with pericarditis secondary to the intramyocardial carcinoid metastases. No surgical options were available. To date the patient has started on peptide receptor radionuclide therapy (PRRT) and will be seen in the cardiology outpatient clinic every 6 months.

## Management

NET patients with CaHD have a significantly decreased life expectancy compared with patients without cardiac involvement [6, 14]. Therefore, early detection and treatment is crucial in preventing right heart failure and improving prognosis. The management of CaHD can be divided into three components: medical therapy, non-cardiac interventions, and cardiac interventions.

### Medical therapy

The main goals in the treatment of patients with carcinoid syndrome are prolongation of progression-free survival, symptom control and subsequent improvement in quality of life. Somatostatin analogues inhibit hormone hypersecretion by binding to somatostatin receptors present on the majority of NET cells [26]. Octreotide [27] and lanreotide [28] have been shown to prolong progression-free survival; however, a significant effect on overall survival has not been demonstrated. In patients with carcinoid syndrome refractory to somatostatin analogues, the novel therapeutic agents telotristat and pasireotide have shown promising results for symptom control [29, 30].

In patients with inoperable or metastatic NETs, PRRT with radiolabelled somatostatin analogues may prove to be beneficial. [31]. PRRT allows for targeted delivery to tumour cells and its effect has been analysed in several large studies [32–34].

More recently, everolimus [35], a mechanistic target of rapamycin (mTOR) inhibitor, and sunitinib [36], an oral vascular endothelial growth factor (VEGF) tyrosine kinase inhibitor, were approved by the US Food and Drug Administration (FDA) for the treatment of advanced pancreatic NETs in 2011 [31]. Despite prolongation of progression-free survival, no increase in overall survival was recorded.

In rapidly progressing pancreatic NETs with a high tumour burden, or in patients with non-pancreatic NETs without other treatment options, chemotherapy is indicated [31, 37], despite the absence of studies showing a beneficial effect on overall survival. Alkylating agents such as streptozocin and temozolomide have been FDA approved although their use is limited due to their toxicity [31]. Temozolomide is deemed less toxic than streptozocin and has shown promising antitumour activity when administered in combination with capecitabine, a prodrug of 5‑fluorouracil [31, 37].

### Non-cardiac interventions

Metastatic disease can be a contraindication for surgical resection of the primary tumour. In contrast, resection of hepatic metastases seems to decrease the risk of cardiac progression and improve prognosis [22]. However, hepatic surgery carries a significant risk of extensive periprocedural bleeding in patients with CaHD, due to the elevated pressures in portal and transhepatic circulation secondary to tricuspid regurgitation. Therefore, cardiac valve surgery is chronologically preferred over hepatic surgery in these patients [22]. Following valve replacement, hepatic resection can be performed relatively safely and is associated with similar outcomes when compared with NET patients without cardiac involvement [38]. Hepatic intra-arterial therapies such as transarterial chemoembolisation and bland embolisation, and selective internal radiotherapy with yttrium-90 microspheres may serve as an alternative to hepatic resection and is predominantly indicated in patients with hepatic metastases [31]. These techniques may induce tumour regression and achieve symptom control. However, limited evidence is available on these interventions and a favourable effect on the progression of CaHD has hence not been demonstrated.

### Cardiac interventions

Upon onset of New York Heart Association (NYHA) heart failure class III or IV symptoms in patients with CaHD, 2‑year survival has been recorded as low as 10% [39]. Valve replacement is the only effective treatment option for symptomatic CaHD patients and is associated with symptomatic improvement [20, 40] and increased survival [20, 39–44]. Recently, 200 CaHD patients (of which 87 underwent cardiac surgery) were analysed and all-cause mortality was assessed [41]. The average age was 63 years and the majority of patients were in NYHA class II or III. Predictors of 10-year all-cause mortality by multivariate Cox proportional hazard analysis were age, urinary 5‑HIAA excretion, moderate or severe RV dilation, and cardiac surgery. Cardiac surgery was associated with a risk reduction of 0.48 (95% CI 0.31 to 0.73, *p* < 0.001). However, these data should be interpreted in light of the non-randomised study design with patients diagnosed in a large time frame from 1981–2000. Importantly, the percentage of patients who underwent cardiac surgery has increased over the years. It is therefore conceivable that the beneficial effect of surgery is influenced by other factors, such as improved medication, experience, and patient selection. A more recent study analysed outcomes after surgical valve replacement in 19 patients [42]. The mean age was 56 years and the average NYHA class was III. A 5-year survival rate of 43% was found. No predictors for mortality were identified although preoperative 5‑HIAA levels were lower in patients who were still alive during data analysis than in those who died (not significant). In a similar study short- and long-term outcomes of CaHD following valve replacement were retrospectively assessed [39]. In total 195 patients were analysed. The mean age was 61 years and 70% of the patients were classified in NYHA class III and IV. All patients underwent tricuspid valve replacement, and 81% pulmonary valve replacement. Survival rates at 1, 5, and 10 years were 69%, 35%, and 24%, respectively. Univariate predictors of overall mortality included age, preoperative creatinine, NYHA class, use of loop diuretics, preoperative chemotherapy, ascites, diabetes mellitus, tobacco use, left-sided valve disease, and right-sided heart size and function.

Patients who are ineligible for cardiac surgery may benefit from pulmonary balloon valvuloplasty. Case studies have been reported where balloon valvuloplasty was performed with major clinical improvements afterwards [45, 46], although relapsing stenosis poses a significant threat [47]. Therefore, surgery should be preferred.

### Perioperative care

Tumour catecholamine release is catalysed by emotional stress, hypercapnia, hypothermia, and hypotension [48]. Furthermore, perioperative vasoactive medications such as epinephrine, norepinephrine, and dopamine are frequently administered to maintain adequate circulation [49], yet are known to provoke carcinoid crisis [48, 49]. Therefore these substances should be administered with caution. At the same time, at the onset of marked hypotension it is difficult to differentiate between carcinoid crisis and the haemodynamic consequences of RV failure. Furthermore, the postoperative course of these patients may also be complicated by bleeding and acute renal dysfunction [50]. Hence the perioperative anaesthetic management of a NET patient with carcinoid syndrome is challenging and requires optimal monitoring. Table 2 provides an overview of the perioperative steps to be taken in anticipation of a carcinoid crisis during surgery.

**Table 2:** Perioperative and hypotension management of cardiac surgery in NET patients

*Perioperative management*
Discontinue ACEi
500 µg octreotide bolus iv preoperatively + iv octreotide pump 2000 µg/24 h
Stop octreotide after detubation if patient is haemodynamically stable
*Hypotension*
NaCl 0.9%
500–1000 µg octreotide bolus + octreotide pump 50–200 µg/h
Inotropes with caution. Only norepinephrine or dopamine

*ACEi* angiotensin converting enzyme inhibitor, *iv* intravenously

### Screening and follow-up

Due to the complexity and rarity of CaHD, patients should be treated in a specialised centre by a multidisciplinary team involving the oncologist, endocrinologist, gastroenterologist, cardiologist, and abdominal and cardiothoracic surgeons [14, 51, 52]. With regards to the indications of screening for CaHD, no consensus has been reached. The UK and Ireland Neuroendocrine Tumour Society (UKINETS) guidelines recommend that all patients with midgut NETs and all patients with carcinoid syndrome should be screened for CaHD, which may include measuring NT-proBNP or echocardiography [52]. Others suggest echocardiography should only be performed in patients with carcinoid syndrome [52] or with elevated NT-proBNP-[51] or 5‑HIAA levels [52]. European Neuroendocrine Society (ENETS) guidelines recommend echocardiographic screening only in patients with carcinoid syndrome or if urinary 5‑HIAA and/or chromogranin A are elevated [19]. An algorithm for the screening for CaHD in patients with metastatic NET with or without carcinoid syndrome has been proposed by others, suggesting annual clinical assessment, TTE and NT-proBNP measurement [25]. In the case of uncertain RV function and suspicion of extracardiac involvement, CMR imaging is recommended. If there is uncertainty regarding valve morphology, transoesophageal echocardiography should be performed. Referral to a cardiologist is recommended on the presence of any of the following criteria: 1) moderate-severe tricuspid/pulmonary regurgitation or stenosis, 2) right heart dilation, 3) RV functional impairment, 4) extracardiac involvement, 5) abnormal tissue Doppler imaging with significantly raised NT-proBNP. In the absence of these criteria TTE should be repeated every 6–12 months. Upon diagnosis of CaHD, the ENETS recommends regular (annual) echocardiographic screening to assess deterioration in heart function [19, 53].

**Fig. 3:**
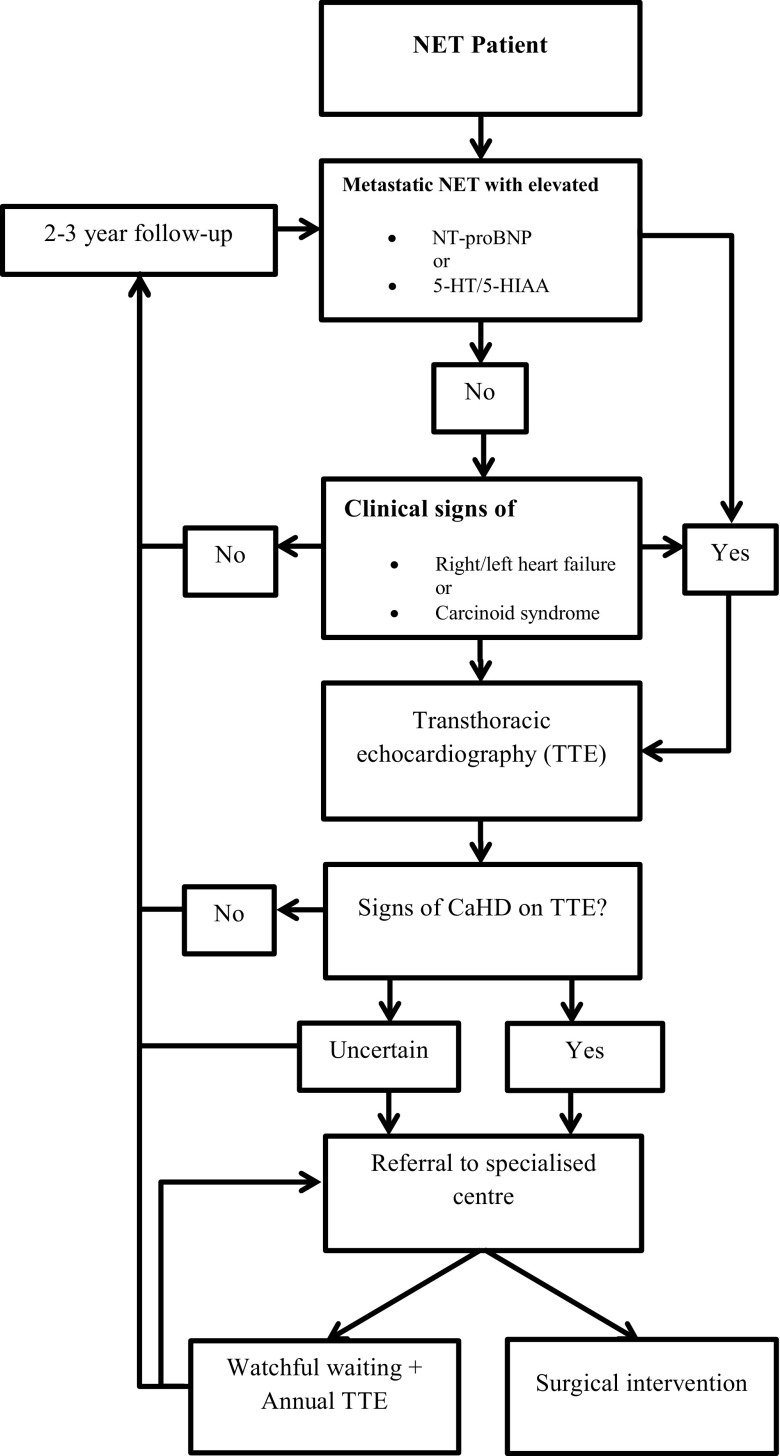
Proposed protocol for screening and referral in CaHD patients. Level of Evidence V. *NET* neuroendocrine tumour, *NT-proBNP* N-terminal pro b‑type natriuretic peptide, *5-HT* 5-hydroxytryptamine,* 5‑HIAA* 5-hydroxyindoleacetic acid, *CaHD* carcinoid heart disease

Here, we propose a protocol to be used as guidance in the screening for CaHD and the referral process (Fig. 3).

In this protocol a liberal screening and referral strategy is implemented to prevent diagnostic and therapeutic delay. Metastatic NET patients with elevated NT-proBNP or serotonin (5-HT/5-HIAA) levels should be assessed with TTE. In the absence of metastatic disease or normal biomarkers, TTE should be performed if clinical signs of right/left heart failure or carcinoid syndrome are present. In the case of confirmed CaHD on TTE, referral to a specialised centre is recommended. A multidisciplinary analysis may either result in surgical intervention, or watchful waiting. In case of the latter, annual TTE is recommended, which may be performed in a non-specialised centre. NET patients without confirmed CaHD on TTE, without clinical signs of right heart failure/carcinoid syndrome or without elevated biomarkers, should be assessed by a cardiologist every 2–3 years.

### Patient selection and timing of cardiac intervention

**Fig. 4:**
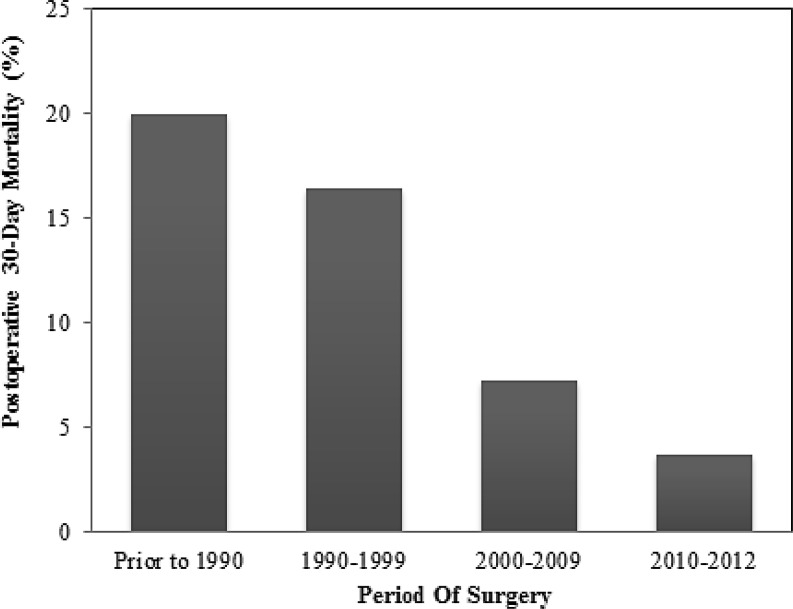
Postoperative 30-day mortality of CaHD patients according to surgical era, adapted from Connolly et al. [39, 40]

Conventionally, valve replacement in CaHD patients has been reserved for patients with symptomatic right heart failure, due to high rates of perioperative mortality in a vulnerable population [53]. However, perioperative mortality has decreased significantly over time [54] and early postoperative mortality has been recorded as low as 10% [41–43], even in patients with NYHA class III and with symptoms of right heart failure [42]. Importantly, 30-day mortality following cardiac surgery has been recorded and a significant decrease from the time period before 1990 (20%) until 2010–2012 (below 5%, Fig. 4) has been observed [39, 40].

This may be explained by a more liberal approach to surgery in recent eras, where both symptomatic and asymptomatic patients with right-sided dysfunction were referred for surgery, as opposed to mainly symptomatic patients. However, other factors may play a role as well, such as improved patient selection, increased experience, progress in oncological management, and advances in surgical techniques [39].

Obviously, the risks of intervention should be weighed against the benefits. The dramatic decrease in perioperative mortality over time raises the question whether a more liberal/ less stringent approach to surgical treatment is indicated. Interestingly, one study found no relation between asymptomatic surgical intervention and long-term survival in multivariate analysis, although this could be explained by the influence of the comorbid malignancy, which may independently affect survival [39]. Despite the decrease in perioperative mortality over time and a trend towards earlier (asymptomatic) surgical intervention [39, 41], limited evidence is available in favour of surgery in asymptomatic patients. Valve replacement in asymptomatic and mildly symptomatic patients (NYHA class I or II) has shown to be associated with a higher postoperative survival rate when compared with severely symptomatic patients (NYHA class III or IV), which advocates early surgical intervention [41]; however, these results should be interpreted with caution as confounding by indication cannot be excluded. Moreover, in this study none of the patients in NYHA class II died in the early postoperative phase. In the absence of clear signs or symptoms of right heart failure, it is challenging to determine which patients ought to be considered for surgery [52]. Biomarkers, and recent echocardiographic techniques such as RV strain assessment may prove to be useful in the early detection of RV dysfunction in these patients [23, 54].

## Conclusion

CaHD has a detrimental effect on prognosis in NET patients. Despite the risks associated with surgery in this population, cardiac intervention has been shown to prolong survival and to increase quality of life. Over time, a trend towards earlier intervention in asymptomatic patients with signs of CaHD has been observed, with increased survival rates and lower perioperative mortality. Although consensus with regards to timing of surgery has not been reached, routine cardiac screening including clinical assessment, biomarkers, and echocardiographic parameters may aid in determining the optimal timing of referral to a specialised centre and subsequent surgical intervention.

## References

[1] Yao JC, Hassan M, Phan A (2008). One hundred years after ‘carcinoid’: Epidemiology of and prognostic factors for neuroendocrine tumors in 35,825 cases in the United States. J Clin Oncol.

[2] Hauso O, Gustafsson BI, Kidd M (2008). Neuroendocrine tumor epidemiology: contrasting Norway and North America. Cancer.

[3] Modlin IM, Kidd M, Latich I (2005). Current status of gastrointestinal carcinoids. Gastroenterology.

[4] Grozinsky-Glasberg S, Grossman AB, Gross DJ (2015). Carcinoid heart disease: from pathophysiology to treatment – ’something in the way it moves’. Neuroendocrinology.

[5] Fox DJ (2004). Carcinoid heart disease: presentation, diagnosis, and management. Heart.

[6] Pellikka PA, Tajik AJ, Khandheria BK (1993). Carcinoid heart disease. Clinical and echocardiographic spectrum in 74 patients. Circulation.

[7] Bhattacharyya S, Toumpanakis C, Chilkunda D (2011). Risk factors for the development and progression of carcinoid heart disease. Am J Cardiol.

[8] Zuetenhorst J, Bonfrer J, Korse C (2003). Carcinoid heart disease: the role of urinary 5‑hydroxyindoleacetic acid excretion and plasma levels of atrial natriuretic peptide, transforming growth factor-beta and fibroblast growth factor. Cancer.

[9] Bhattacharyya S, Toumpanakis C, Caplin ME (2008). Analysis of 150 patients with carcinoid syndrome seen in a single year at one institution in the first decade of the twenty-first century. Am J Cardiol.

[10] Pandya UH, Pellikka PA, Enriquez-Sarano M (2002). Metastatic carcinoid tumor to the heart: Echocardiographic-pathologic study of 11 patients. J Am Coll Cardiol.

[11] Greminger P, Hess OM, Müller AE (1991). Bronchial neuroendocrine (carcinoid) tumor causing unilateral left-sided carcinoid heart disease. Klin Wochenschr.

[12] Marupakula V, Vinales KL, Najib MQ (2011). Occurrence of left-sided heart valve involvement before right-sided heart valve involvement in carcinoid heart disease. Eur J Echocardiogr.

[13] Møller JE, Connolly HM, Rubin J (2003). Factors associated with progression of carcinoid heart disease. N Engl J Med.

[14] Dobson R, Burgess MI, Pritchard DM (2014). The clinical presentation and management of carcinoid heart disease. Int J Cardiol.

[15] Ross EM, Roberts WC (1985). The carcinoid syndrome: Comparison of 21 necropsy subjects with carcinoid heart disease to 15 necropsy subjects without carcinoid heart disease. Am J Med.

[16] Bhattacharyya S, Toumpanakis C, Caplin ME (2008). Usefulness of N‑terminal pro-brain Natriuretic peptide as a Biomarker of the presence of Carcinoid heart disease. Am J Cardiol.

[17] Vinik AI, Woltering EA, Warner RR (2010). NANETS consensus guidelines for the diagnosis of neuroendocrine tumor. Pancreas.

[18] Dobson R, Burgess MI, Valle JW (2014). Serial surveillance of carcinoid heart disease: factors associated with echocardiographic progression and mortality. Br J Cancer.

[19] Pape UF, Perren A, Niederle B (2012). ENETS consensus guidelines for the management of patients with neuroendocrine neoplasms from the jejuno-ileum and the appendix including goblet cell carcinomas. Neuroendocrinology.

[20] Robiolio PA, Rigolin VH, Harrison JK (1995). Predictors of outcome of tricuspid valve replacement in carcinoid heart disease. Am J Cardiol.

[21] Plöckinger U, Gustafsson B, Ivan D (2009). ENETS consensus guidelines for the standards of care in neuroendocrine tumors: Echocardiography. Neuroendocrinology.

[22] Luis SA, Pellikka PA (2016). Carcinoid heart disease: diagnosis and management. Best Pract Res Clin Endocrinol Metab.

[23] Haugaa KH, Bergestuen DS, Sahakyan LG (2011). Evaluation of right ventricular dysfunction by myocardial strain echocardiography in patients with intestinal carcinoid disease. J Am Soc Echocardiogr.

[24] Mansencal N, McKenna WJ, Mitry E (2010). Comparison of prognostic value of tissue doppler imaging in Carcinoid heart disease versus the value in patients with the Carcinoid syndrome but without Carcinoid heart disease. Am J Cardiol.

[25] Dobson R, Cuthbertson DJ, Burgess MI (2013). The optimal use of cardiac imaging in the quantification of carcinoid heart disease. Endocr Relat Cancer.

[26] Bousquet C, Lasfargues C, Chalabi M (2012). Current scientific rationale for the use of somatostatin analogs and mTOR inhibitors in neuroendocrine tumor therapy. J Clin Endocrinol Metab.

[27] Rinke A, Müller HH, Schade-Brittinger C (2009). Placebo-controlled, double-blind, prospective, randomized study on the effect of octreotide LAR in the control of tumor growth in patients with metastatic neuroendocrine midgut tumors: A report from the PROMID study group. J Clin Oncol.

[28] Caplin ME, Pavel M, Ćwikła JB (2014). Lanreotide in metastatic Enteropancreatic Neuroendocrine tumors. N Engl J Med.

[29] Kulke MH, O’Dorisio T, Phan A (2014). Telotristat etiprate, a novel serotonin synthesis inhibitor, in patients with carcinoid syndrome and diarrhea not adequately controlled by octreotide. Endocr Relat Cancer.

[30] Wolin EM, Jarzab B, Eriksson B (2015). Phase III study of pasireotide long-acting release in patients with metastatic neuroendocrine tumors and carcinoid symptoms refractory to available somatostatin analogues. Drug Des Devel Ther.

[31] Kunz PL (2015). Carcinoid and neuroendocrine tumors: building on success. J Clin Oncol.

[32] Bushnell DL, O’Dorisio TM, O’Dorisio MS (2010). 90Y-edotreotide for metastatic carcinoid refractory to octreotide. J Clin Oncol.

[33] Kwekkeboom DJ, De Herder WW, Kam BL (2008). Treatment with the radiolabeled somatostatin analog [177Lu- DOTA0,Tyr3]octreotate: Toxicity, efficacy, and survival. J Clin Oncol.

[34] Strosberg J, El-Haddad G, Wolin E (2017). Phase 3 Trial of ^177^ Lu-Dotatate for Midgut Neuroendocrine Tumors. N Engl J Med.

[35] Yao JC, Pavel M, Lombard-Bohas C (2016). Everolimus for the treatment of advanced pancreatic Neuroendocrine tumors: overall survival and circulating biomarkers from the randomized, phase III RADIANT-3 study. J Clin Oncol.

[36] Raymong E, Dahan L, Raoul J (2011). Sunitinib Malate for the treatment of pancreatic Neuroendocrine tumors. N Engl J Med.

[37] Pavel M, Baudin E, Couvelard A (2012). ENETS consensus guidelines for the management of patients with liver and other distant metastases from neuroendocrine neoplasms of foregut, midgut, hindgut, and unknown primary. Neuroendocrinology.

[38] Lillegard JB, Fisher JE, McKenzie TJ (2011). Hepatic resection for the carcinoid syndrome in patients with severe carcinoid heart disease: does valve replacement permit safe hepatic resection?. J Am Coll Surg.

[39] Connolly HM, Schaff HV, Abel MD (2015). Early and late outcomes of surgical treatment in carcinoid heart disease. J Am Coll Cardiol.

[40] Connolly HM, Nishimura RA, Smith HC (1995). Outcome of cardiac surgery for carcinoid heart disease. J Am Coll Cardiol.

[41] Møller JE, Pellikka PA, Bernheim AM (2005). Prognosis of carcinoid heart disease: analysis of 200 cases over two decades. Circulation.

[42] Mokhles P, van Herwerden LA, de Jong PL (2012). Carcinoid heart disease: outcomes after surgical valve replacement. Eur J Cardiothorac Surg.

[43] Castillo JG, Filsoufi F, Rahmanian PB (2008). Early and late results of valvular surgery for Carcinoid heart disease. J Am Coll Cardiol.

[44] Edwards NC, Yuan M, Nolan O (2016). Effect of valvular surgery in carcinoid heart disease: An observational cohort study. J Clin Endocrinol Metab.

[45] Obel O, Coltart DJ, Signy M (2000). Balloon pulmonary valvuloplasty in carcinoid syndrome. Heart.

[46] Carrilho-Ferreira P, Silva D, Almeida AG (2013). Carcinoid heart disease: outcome after balloon pulmonary Valvuloplasty. Can J Cardiol.

[47] Grant SC, Scarffe JH, Levy RD (1992). Failure of balloon dilatation of the pulmonary valve in carcinoid pulmonary stenosis. Br Heart J.

[48] Castillo JG, Silvay G, Solís J (2013). Current concepts in diagnosis and perioperative management of carcinoid heart disease. Semin Cardiothorac Vasc Anesth.

[49] Weingarten TN, Abel MD, Connolly HM (2007). Intraoperative management of patients with carcinoid heart disease having valvular surgery: A review of one hundred consecutive cases. Anesth Analg.

[50] Regner KR, Connolly HM, Schaff HV (2005). Acute renal failure after cardiac surgery for carcinoid heart disease: Incidence, risk factors, and prognosis. Am J Kidney Dis.

[51] Silaschi M, Barr J, Chaubey S (2016). Optimized outcomes using a standardized approach for treatment of patients with Carcinoid heart disease. Neuroendocrinology.

[52] Mota JM, Sousa LG, Riechelmann RP (2016). Complications from carcinoid syndrome: review of the current evidence. Ecancermedicalscience.

[53] Ramage JK, Ahmed A, Ardill J (2012). Guidelines for the management of gastroenteropancreatic neuroendocrine (including carcinoid) tumours (NETs). Gut.

[54] Warner RRP, Castillo JG (2015). Carcinoid heart disease. J Am Coll Cardiol.

